# Two sporadic patients of Perrault syndrome with ovarian dysgenesis and sensorineural deafness

**DOI:** 10.1186/1687-9856-2013-S1-P81

**Published:** 2013-10-03

**Authors:** Young-Lim Shin

**Affiliations:** 1 Pediatrics, Soonchunhyang University Bucheon Hospital, South Korea

## 

Perrault syndrome is an autosomal recessive disorder characterized by sensorineural deafness and ovarian dysgenesis. Some patients also have neurologic abnormalities, including cerebellar ataxia, nystagmus, polyneuropathy and mild mental retardation. The syndrome is known to be caused by mutations in *HSD17B4* or *HARS2* until now but few patients were reported. We report on two sporadic Korean patients of Perrault syndrome with ovarian dysgenesis and sensorineural deafness.

Patient 1 : A 14 year-6 month female patient presented with delayed puberty. She had bilateral sensorineural hearing impairment. Her neurological findings were normal and She had no mental retardation. She had normal karyotype (46, XX) and hypergonadotropic hypogonadism. Pelvic ultrasound showed a small uterus and ovaries. Brain MRI was normal.

Patient 2 : A 16 year-11 month female presented with delayed puberty and neurologic manifestations. She had ataxia, dyspraxia and weakness. She had no mental retardation and brain MRI was normal. She had multiple Café au lait spots and long slender fingers but no Marfanoid features. Laboratory tests revealed hypergonadotropic hypogonadism and normal female karyotype (46, XX). Pelvic ultrasound examination showed a hypoplastic uterus and small ovaries.

Because Perrault syndrome has clinical variability and genetically heterogeneous, we have to inspect additional findings and neurological abnormalities. And further studies will be required to ascertain the common causative mutation of this syndrome.

